# Estimation of Sialic Acid and IL10 Levels in Stage 1 and 2 Periodontitis Patients

**DOI:** 10.1155/2019/2917124

**Published:** 2019-11-29

**Authors:** Sudhir Rama Varma, Biju Thomas, Amitha Ramesh, Suchetha Kumari, Shahira, Zainab Abdullah Muhammad Al Saadi, Rabia Asif Mahmood

**Affiliations:** ^1^Department of Periodontics, University of Science and Technology of Fujairah, Fujairah, UAE; ^2^Department of Periodontics, AB Shetty Memorial Institute of Dental Sciences, Mangaluru, Karnataka, India; ^3^Department of Biochemistry, K. S. Hegde Medical Academy, Deralakatte, Mangaluru, Karnataka, India; ^4^College of Dentistry, Ajman University of Science and Technology, Ajman, UAE

## Abstract

**Objective:**

The role of biomarkers in staging and grading periodontal disease has become detrimental in relation to the overall treatment plan. This study aimed at evaluating and comparing the role of sialic acid and IL10 in the early and moderate stages of periodontitis.

**Materials and Methods:**

Patients were selected according to the assessment of pocket depth and radiographic bone loss. Bone loss was calculated as <15% for stage 1 and 15–33% for stage 2. Salivary samples were collected using spit technique 2 hrs post consumption of food. The unstimulated saliva was collected in a sterile graduated container every minute for 5–8 minutes. IL10 estimation was done using ELISA, and sialic acid estimation was done using the diphenylamine method. The variables for the three groups were assessed using ANOVA, and intragroup comparisons for quantitative data were evaluated using the post hoc Bonferroni test (*P* < 0.05).

**Results:**

On comparing sialic acid levels among the three groups, stage 2 showed the highest mean (8.61) compared with the other two groups and was highly significant (*P* < 0.001). On the contrary, IL10 when compared to stage 1 and 2 periodontitis revealed insignificant change.

**Conclusion:**

The value of IL10 was higher as patients progressed from health to periodontitis.

## 1. Introduction

Periodontitis is a multifactorial disease which is inflammatory in origin. It results due to a dysbiosis between host immunological and bacterial cytokine release aggravated by local, systemic, and environmental factors [1]. The immunomodulatory mechanisms resulting due to this interaction result in the release of proinflammatory and anti-inflammatory cytokines that play a detrimental role in the overall progress of the disease [2]. This further leads to destruction of periodontal soft tissues and in course of time attachment loss and increased osteoclastic activity, leading to alveolar bone loss [3]. Periodontitis has been classified over the years on various factors such as extent, distribution, etiology, pathogenesis, and immunological factors. The 2017 workshop classification revamped the old classification according to the pathophysiology of the disease and due to the results of various studies conducted over a period of 19 years. New terminologies were proposed, and the highlight of the classification was that the terms chronic and aggressive were removed [4, 5]. The classification was proposed on a multidimensional staging and grading criteria that could be modified over time as new information surfaces [6, 7]. Furthermore, Tonetti et al. proposed the staging and grading for periodontal diseases according to a framework replicating the oncology staging criteria proposed goals for the staging and grading for periodontitis patients [7]. The goals were to classify the severity and extent of periodontal disease and also to determine the complexity of the periodontal condition. The grading for a patient with a periodontal condition was to determine the risk in a longitudinal time frame, i.e., risk in future and also to follow-up on the impact of systemic factors with relation to periodontal diseases. The staging for periodontal disease resulted in stage 1 and 2 for mild and moderate periodontitis and 3 and 4 for severe and very severe periodontitis.

Periodontal disease progression leads to an increase in reactive oxygen species (ROS) and enzymes which is proteolytic in nature that result in host tissue damage and release of increased levels of biomarkers [8]. The recent development in biochemical tests have resulted in identifying specific biomarkers that are more or less associated with the disease process. The use of GCF, plaque, saliva, and serum has helped researchers in isolating these proteins from possible “high risk” patients [9].

Sialic acid and IL10 play an important role in identifying inflammation that is progressing to high risk. Sialic acid is associated with disease and is a proinflammatory enzyme, and IL10 is an anti-inflammatory cytokine which is predominantly seen in both health and disease.

An important function of sialic acid is regulation of host innate immunity [10, 11]. It is an acetylated derivative of neuraminic acid; the generic term is sialic acid. Biofilm-based microorganisms absorb sialic acid to evade innate immune response triggered by the host. Release of SA by sialidase enzyme of bacteria or host-derived neuraminidase results in collateral damage to the host tissues [12].

IL10 is an anti-inflammatory cytokine that stimulates antibody production and is seen in both health and disease [13]. Though in stage 3 periodontitis the severity of periodontitis correlates with bone loss, the presence of IL10 is reduced in such a situation [14]. It regulates the production of proinflammatory cytokines such as IL1, IL2, and Il6. Data in the literature are contradictory to the presence of IL10 and its variations with relation to periodontal disease [15].

The aim of the present study is to evaluate and compare the presence of sialic acid (SA) and IL10 in the early stage (stage 1) and moderate stage (stage 2) of periodontal disease.

## 2. Materials and Methods

### 2.1. Study Population

This interventional study was a randomized, multicenter trial. The duration of the study was 5 months, from October 2018 to February 2019. 75 patients attending the outpatient clinic of the department of periodontics, AB Shetty Dental College and Ajman University, Fujairah campus were recruited for the study. Written informed consent had been taken for those who agreed to participate. The research protocol was approved by the ethical committee (UGD-H-18-10-31-10). It is registered in the ClinicalTrials.gov ID: NCT03775967. The research manuscript follows consolidated standards of reporting trials (CONSORT) guidelines as well as the Helsinki Declaration for human research as revised in 2013.

Inclusion criteria: patients in the age group of 21–75 years having stage 1 and stage 2 periodontitis without systemic conditions. Healthy controls were selected based on no gingivitis and periodontal findings and systemically stable.

Exclusion criteria: patients having systemic conditions, have taken medications in the past 3 months, or have had periodontal therapy in the past 3 months.

### 2.2. Oral Examination

Stage 1 and 2 periodontitis were selected according to the staging proposed by Tonetti et al. [13]. Pocket depth was measured using Ramfjord teeth as there was a positive correlation between the index teeth and pocket depth as concluded by Mumghamba et al. [16]. Radiographic bone loss using the long cone technique was calculated as <15% for stage 1 and between 15 and 33% for stage 2 as proposed by the AAP 2017 classification [6]; probing pocket depth was measured by using a periodontal probe (Williams probe, Hu-Friedy, Chicago, IL, USA), and six sites were selected for each index teeth.

### 2.3. Biochemical Analysis

All salivary samples were collected from the subjects using the spit technique 2 h after consumption of food. Unstimulated saliva was collected by asking the subject to spit in a sterile graduated container every minute for 5–8 min. The collected sample was centrifuged at 3000 rpm for 10 min, and the supernatant was collected and stored at −20°C to obtain a clear supernatant.

IL10 estimation was performed using an ELISA kit (Elispot kit, ab46601, Abcam, USA).

Sialic acid estimation was performed by the diphenylamine method. SA was made to react with diphenylamine producing a purple color which was then quantitatively estimated using a spectrophotometer of 530 nm [17].

### 2.4. Statistical Analysis

Sample size was calculated keeping the confidence level at 95%, with a confidence interval at 5%. The population prevalence was assumed as 90 for a period of five months. The sample size obtained was 73 which was rounded up significantly to 75. The variables for the three groups were assessed using ANOVA (Analysis of Variance). The post hoc Bonferroni test was used for quantitative data comparison (intragroup comparison) of all variables. Analysis was done using SPSS version 20.0 (IBM SPSS Statistics Inc., Chicago, Illinois, USA) windows program. Level of significance was set at *P* < 0.05.

## 3. Results

Comparison of sialic acid among the three groups,namely, (Table 1) health and stage 1 and stage 2 periodontitis using ANOVA showed stage 2 with the highest mean level of sialic acid (8.61) as compared with stage 1 (8.55) and health (0.9) groups, which showed a highly significant result *P* < 0.001. Comparing stage 1 and stage 2 periodontitis, slight changes among sialic acid levels were seen.

Evaluating intragroup comparison using the post hoc Bonferroni test (Table 2) for only the health group showed statistically highly significant results against stage 1 and 2 periodontitis (*P* < 0.001). Stage 1 and 2 periodontitis showed slight changes among sialic acid levels and indicated nonsignificant results when compared between the two.

Comparing IL10 among health and stage 1 and 2 periodontitis groups (Table 3), stage 2 periodontitis showed the highest mean level of IL10 (133.05) as compared with stage 1 (129.26) and health (93.92) groups which showed highly significant results. IL10 levels among stage 1 and 2 periodontitis cases revealed a minimal change.

The control group (health) showed highly significant results (*P* < 0.001) compared with stage 1 and 2 periodontitis patients (Table 4). Stage 1 and 2 showed nominal changes among IL10 levels and indicated nonsignificant results during comparison.

## 4. Discussion

One of the markers in the acute phase inflammation is sialic acid [18]. It is associated with various commonly seen systemic disorders such as cardiovascular disease, diabetes mellitus, pulmonary disorders, and liver dysfunctions [19, 20]. The presence of increased sialic acid in periodontitis patients was earlier proposed by Shinohara et al. and can bring about a distinct boundary between health and disease [21]. Sialic acid levels were found to be higher among smokers which was associated with a release of free radicals [22]. One of the mechanisms proposed by Eguchi et al. is the release of free radicals which results due to the hydrolysis of the glycosidic linkage of the terminal end of the sialic acid molecule [23]. IL10, being an anti-inflammatory cytokine, regulates the activity of proinflammatory cytokines as suggested in various studies [24–26]. It is reported to be reduced in active periodontal disease such as localized aggressive periodontitis, where the predominant microorganism is *Aggregatibacter actinomycetemcomitans* [15]. In our present study, we estimated levels of sialic acid and IL10 in saliva from healthy and stage 1 and stage 2 periodontitis patients. There was statistically highly significant (*P* < 0.001) reading between healthy controls and stage 1 and 2 periodontitis patients on comparing salivary sialic acid levels. Though the comparison between stage 1 and 2 periodontitis was significantly lesser, there was a positive correlation between health and disease. This was further in accordance with other studies by Davis et al. [27–29]. This can also be further validated by a study by Jawazaly where a direct association between periodontal disease and increased concentrations of sialic acid levels was seen [28]. Shetty and Pattabiraman also found a positive correlation between sialic acid levels and gingivitis [30]. The results of IL10 in our study were significantly high comparing health and stage 1 and 2 periodontitis patients. This is in agreement to a study by Lappin et al. [31]. Studies with a similar result show that higher titer of IL10 was seen between patients having aggressive periodontitis and healthy controls, though in this study the means did not differ [32, 33]. This was contrary to another study where the levels of IL10 showed a statistically significant decrease when compared with health [34]. The study by Giannobile et al. showed a downward slope of IL10 in periodontal disease indicating lower levels [35]. The study evaluated that IL10 obtained from GCF was found to contain higher levels of IL10 with relation to a specific IL6 genotype [36]. This could possibly be due to the inflammatory component which has been regulated by IL10. Furthermore, a study by Hannum et al. has found lack of association with relation to IL10 gene polymorphism and stage 1 and 2 periodontal disease [37]. A possible reason for these conflicting results with relation to IL10 could be ethnic differences in the distribution of IL10 polymorphisms, as seen in various studies reporting varied levels of IL10 and its association in stage 1 and 2 periodontitis cases and in aggressive variety [38, 39]. The results from our study highlights the fact that IL10, though categorized as an anti-inflammatory cytokine, was found in increased values as the levels of disease activity increased. The limitations in our study were the small sample size, and therefore our results need to be validated in larger sample size and also tested in population from different ethnicities.

## 5. Conclusion

Based on the findings of the current study, increased sialic acid levels had a positive correlation from health to disease. The value of IL10 in our study was higher from health to periodontitis.

The reason is not fully understood, which does not correlate with results from some of the studies. Studies evaluating IL10 with respect to GCF, serum, and saliva need to be compared in a larger sample size. More genetic studies with relation to different ethnicities are needed to be done in order to corroborate the results.

## Figures and Tables

**Table 1:** ANOVA comparison of sialic acid among groups.

	Mean	Std. deviation	Minimum	Maximum	*P* value
Stage 1	8.55	4.13	1.81	18.18	0.001 (S)
Stage 2	8.61	3.42	4.00	15.27	
Stage 3	0.9	0.37	0.32	2.30	

*P* value *P* < 0.001 highly significant.

**Table 2:** Post hoc Bonferroni test for intragroup comparison.

		Mean differences	*P* value
Stage 1	Stage 2	−0.059	1.000
	Health	7.65	0.001 (S)
Stage 2	Stage 1	0.059	1.000
	Health	7.71	0.001 (S)
Health	Stage 1	−7.65	0.001 (S)
	Stage 2	−7.71	0.001 (S)

*P* < 0.001 is highly significant.

**Table 3:** Comparison of IL10 among groups.

	Mean	Std. deviation	Minimum	Maximum	*P* value
Stage 1	129.26	31.706	44.87	195.23	0.001 (S)
Stage 2	133.05	30.08	34.90	182.16	
Health	93.92	6.56	80.00	110.00	

*P* < 0.001 is highly significant.

**Table 4:** Post hoc Bonferroni test for intragroup comparison.

		Mean differences	*P* value
Stage 1	Stage 2	−3.79	1.000
	Health	35.34	0.001 (S)
Stage 2	Stage 1	3.79	1.000
	Health	39.13	0.001 (S)
Health	Stage 1	−35.34	0.001 (S)
	Stage 2	−39.13	0.001 (S)

*P* < 0.001 is highly significant.

## Data Availability

The data used to support the findings of this study are included in the article.
